# An agent-based model simulation of influenza interactions at the host level: insight into the influenza-related burden of pneumococcal infections

**DOI:** 10.1186/s12879-017-2464-z

**Published:** 2017-06-02

**Authors:** Hélène Arduin, Matthieu Domenech de Cellès, Didier Guillemot, Laurence Watier, Lulla Opatowski

**Affiliations:** Biostatistics, Biomathematics, Pharmacoepidemiology and Infectious Diseases, UMR1181 - Université de Versailles Saint Quentin en Yvelines, Inserm, Institut Pasteur, B2PHI Unit – Institut Pasteur, 25 rue du Docteur Roux, 75724 Paris Cedex 15, France

**Keywords:** Agent-based model, Influenza, Between-pathogens interaction, Interference, Transmission dynamics, Pneumococcus, Simulation, Burden, Mathematical model

## Abstract

**Background:**

Host-level influenza virus–respiratory pathogen interactions are often reported. Although the exact biological mechanisms involved remain unelucidated, secondary bacterial infections are known to account for a large part of the influenza-associated burden, during seasonal and pandemic outbreaks. Those interactions probably impact the microorganisms’ transmission dynamics and the influenza public health toll. Mathematical models have been widely used to examine influenza epidemics and the public health impact of control measures. However, most influenza models overlooked interaction phenomena and ignored other co-circulating pathogens.

**Methods:**

Herein, we describe a novel agent-based model (ABM) of influenza transmission during interaction with another respiratory pathogen. The interacting microorganism can persist in the population year round (endemic type, e.g. respiratory bacteria) or cause short-term annual outbreaks (epidemic type, e.g. winter respiratory viruses). The agent-based framework enables precise formalization of the pathogens’ natural histories and complex within-host phenomena. As a case study, this ABM is applied to the well-known influenza virus–pneumococcus interaction, for which several biological mechanisms have been proposed. Different mechanistic hypotheses of interaction are simulated and the resulting virus-induced pneumococcal infection (PI) burden is assessed.

**Results:**

This ABM generates realistic data for both pathogens in terms of weekly incidences of PI cases, carriage rates, epidemic size and epidemic timing. Notably, distinct interaction hypotheses resulted in different transmission patterns and led to wide variations of the associated PI burden. Interaction strength was also of paramount importance: when influenza increased pneumococcus acquisition, 4–27% of the PI burden during the influenza season was attributable to influenza depending on the interaction strength.

**Conclusions:**

This open-source ABM provides new opportunities to investigate influenza interactions from a theoretical point of view and could easily be extended to other pathogens. It provides a unique framework to generate in silico data for different scenarios and thereby test mechanistic hypotheses.

**Electronic supplementary material:**

The online version of this article (doi:10.1186/s12879-017-2464-z) contains supplementary material, which is available to authorized users.

## Background

Influenza is a respiratory virus that imposes a heavy burden on human populations by causing millions of severe illness cases and deaths worldwide every year [1], despite the development of public health policies to limit its propagation. The virus infects the human respiratory tract where other species of bacteria or viruses cohabit. In recent years, interest has grown in the potential role of pathogen–pathogen interactions for host respiratory tract infection or colonization. These interactions have been investigated from different biological, clinical and epidemiological perspectives [2–4]. Several pathogens are thought to interfere with influenza viruses: respiratory syncytial virus [5], rhinovirus [5, 6], *Streptococcus pneumoniae* [2, 7, 8], *Haemophilus influenza* [9], *Staphylococcus aureus* [10] etc. Even though the biological mechanisms governing those interactions are not well known, many hypotheses have been advanced. The presence of a second pathogen has been postulated to alter some pathogens’ natural cycles within the host or to modify their transmission patterns [2, 7, 11, 12]. If they indeed exist, these interactions might impact the pathogens’ transmission dynamics in human populations and their burden in terms of public health.

A long-studied interaction among respiratory pathogens is that between influenza viruses and *Streptococcus pneumoniae* (pneumococcus) [2, 8, 13]. Influenza usually infects the human upper respiratory tract but can also spread to the lower respiratory tract, causing more severe illness [14, 15]. Pneumococcus is a very common bacterial species of the human upper respiratory tract [16], that asymptomatically colonizes the nasopharynx of individuals and is naturally cleared after a few weeks, depending on age [17, 18]. When the bacteria invades the surrounding tissues or the bloodstream, various bacterial infections can occur, ranging from mild otitis media to more severe pneumonia or meningitis [19]. A synergistic relationship between these two pathogens has been suggested [8], with influenza infections thought to increase the risk of severe secondary pneumococcal infections (PI) [7, 8, 20]. However, the interaction mechanisms and consequences in terms of morbidity or mortality, and, more generally, the public health burden, are still very poorly known.

Mathematical modeling is a powerful tool to explore the epidemiology and spread of infectious diseases [21–23]. Historically, most published models have been based on a compartmental approach formalizing the transmission of a given pathogen of interest, while neglecting all the other microorganisms simultaneously colonizing or infecting the population. In those models, pathogens are assumed to infect populations in an independent manner [24–26]. In recent years, the compartmental approach has proven very useful to assess biological hypotheses exploring pathogen interactions and their implications in terms of public health [27–29]. However those models become very complex when trying to model precisely the phenomena occurring at the individual level. In contrast, the agent-based model (ABM) framework is particularly appropriate in this context. Processes are defined at the individual level, allowing a detailed definition of each pathogen’s natural history and the complexity of within-host phenomena. For example, the exact interaction timeframe can be precisely set in an ABM, whereas it is much more difficult to do so with compartmental models.

Herein, we describe a novel individual-based simulator of the co-circulation of influenza virus (henceforth referred to as influenza) in interaction with another respiratory pathogen in a virtual population of humans, the Simulator of Flu in Interaction (SimFI) framework. We used this framework to explore the particular context of influenza and pneumococcus co-circulation. Based on simulated data, we assessed the potential burden of PI cases attributable to influenza, under different interaction hypotheses.

## Model and methods

We developed a stochastic ABM, in which each human individual is specifically modeled and his/her successive infectious states recorded. The model is applied to simulate influenza and a second pathogen, which can be either epidemic (causing annual outbreaks but absent most of the year) or endemic (present in the population throughout the year). The between-pathogen interactions and their consequences can vary according to the chosen pathogen.

We used the NetLogo multi-agent programmable modeling environment for the implementation [30]. The model is freely available on the following website http://b2phi.inserm.fr/#/resources/71/NetLogo-SimFI-model.

### Global overview

Individuals are characterized by several variables monitoring their location, age and infectious state concerning the two pathogens. The simulation time step is the day. On each day of the simulation individuals move in the simulated world and come into contact with other people. From those contacts, the transmission of one or both pathogens is possible and will result in reevaluations of the variables describing infectious status. Here years define epidemic years, starting on October 1st and ending on September 30th.

#### Simulation set-up

The model is artificially spatially explicit so that the individuals can move, in what the NetLogo software refers to as a “world”, which is a torus divided into patches. Two persons are considered to be in contact if they coincide on the same patch at a given time. The dimension of the patches – and thus of the world – is set in order to have, on average, 13 contacts per person and per day as reported by the Polymod study and its French equivalent [31, 32]. The individuals move in this world each day in a randomly chosen direction at a distance ranging from 1 to 10 patches. The individuals are initially healthy and uniformly distributed at random around the world, with uniformly distributed ages. By default, birth and death rates are fixed in order to keep the population size constant at 100,000 individuals.

#### Natural histories of the pathogens

**Fig. 1:**
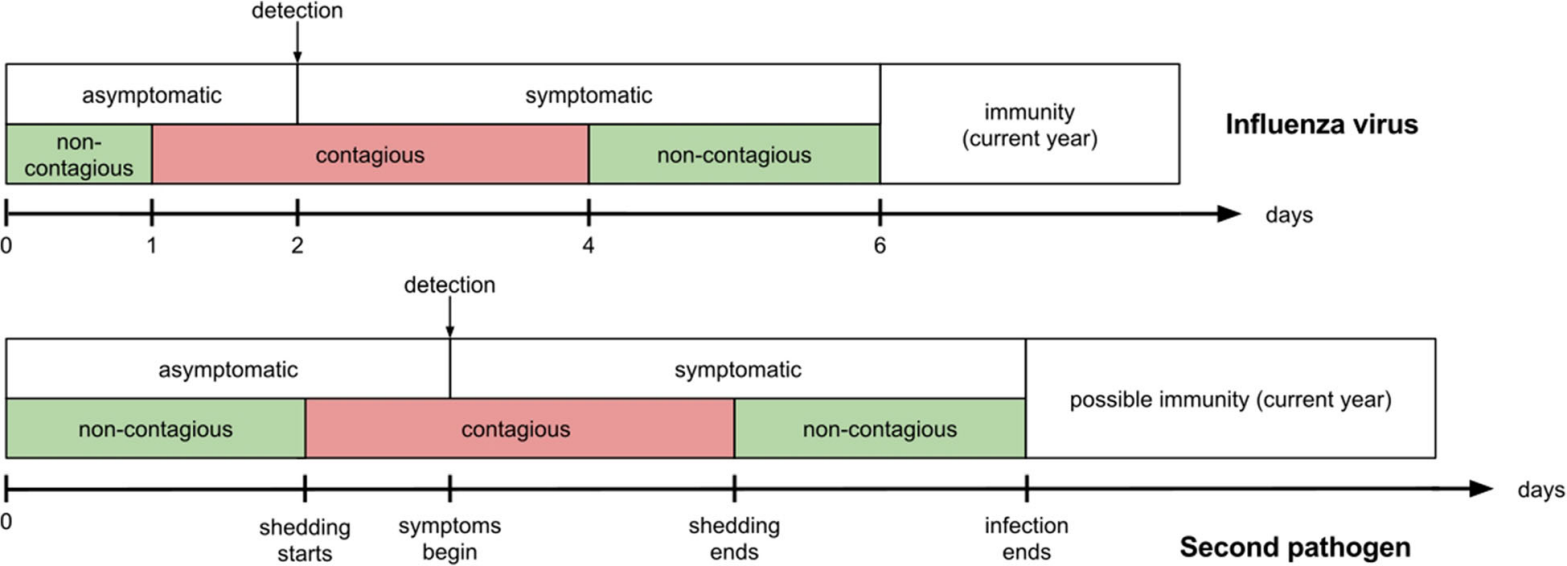
Natural histories of the two simulated pathogens. Clinical status (upper rectangles) and infectious status (green for non-contagious, red for contagious) are shown for influenza (top timeline) and the second interacting pathogen (bottom timeline)

Figure 1 illustrates the natural histories of the pathogens. At any given time, all individuals can acquire one or both circulating pathogens. During the simulation, when a new individual enters a new infectious state, the duration for which he/she will remain in this state is drawn from a Gamma distribution, with a mean value set by the user.

### Influenza implementation

#### Natural history

Once an individual is infected with an influenza virus, the incubation period is assumed to last an average of 2 days [33, 34], followed by a symptomatic phase lasting an average of 4 days [26, 27, 32, 33]. During these two stages, we assumed that the individual would be contagious from day 2 post-virus infection until 2 days after the symptoms began [27, 33, 34]. Once the symptomatic period is over, the individual is assumed to be immune to the current virus. Default values for the influenza cycle can be found in Fig. 1 timeline and Table 1.

**Table 1:** Main model parameters and their default values

Description	Default value	Rationale
Population size	100,000 persons	chosen for computational purposes
Influenza virus		
Transmission-probability rate	*Ν*(mean 0.03; SD^∗^ 0.001) per contact-day	calibrated to match French influenza-like illnesses data [35]
Reporting probability of symptomatic cases	20%	
Initial percentage of immune people each year	23%	
Incubation period	*Γ*(mean 2; var 0.1)	[33, 34]
Symptomatic period	*Γ*(mean 4; var 1)	[26, 27, 34, 49]
Shedding period	(1 day after asymptomatic state onset; 2 days after symptomatic state onset)	[27, 33, 34]
Second pathogen (pneumococcus example default values)		
Asymptomatic period duration	*Γ*(mean 21; var 25)	[18, 37]
Infection-probability rate	4.2e-5 per day	calibrated to obtain an average annual incidence of 220 PI cases per 100,000 [38]
Symptomatic case-reporting probability	100%	all PI cases are assumed to be reported
Symptomatic period duration	*Γ*(mean 12; var 16)	[38]
Shedding onset in the asymptomatic phase	Day 0	[17, 39, 40]
Shedding end in the symptomatic phase	Day 2	due to severity, PI cases are assumed to be isolated after 2 days of symptoms
Acquisition interaction (α_12_, α_21_)	(1, 1)	to be varied
Transmission interaction (θ_12_, θ_21_)	(1, 1)	to be varied
Endemic case: specific parameters		
Carriage rate in the population (% of asymptomatic individuals)	20%	[17, 39, 40]
Immunity-period duration (immunity reinitialized at the end of every year)	300 days	no immunity assumed for pneumococcus
Pathogenicity interaction (π_12_)	1	to be varied
Epidemic case: specific parameters		
Beginning of the 2nd pathogen epidemic	*U*(30 – 60) days after influenza epidemic onset	can be varied according to the chosen pathogen
No. of cases when the 2nd pathogen epidemic starts	*U*(20 – 30)	
Initial percentage of immune people	25%	
Cross-immunity interaction (μ_12_, μ_21_)	(1, 1)	to be varied

^*^SD: standard deviation.

#### Influenza set-up

On the first day of each epidemiological year, the characteristics of the coming influenza outbreak are set. The influenza-associated infectiousness probability is drawn from a normal probability distribution with a mean value set by the user (3.3% per contact-day by default). A portion of randomly chosen individuals is immunized against the coming influenza virus (default at 23%) to take into account natural or acquired immunity in the population. The importation date of the first cases is randomly drawn from a gamma distribution of mean 71 and standard deviation 28, and the number of imported cases is randomly chosen between 20 and 30. Every individual has a 20% probability of being reported when they enter the symptomatic phase. Default values were chosen to obtain incidence series comparable to French surveillance data from the Sentinel Network [35].

### Second pathogen implementation

#### Natural history

Individuals can acquire the second pathogen in a similar way as they acquire influenza, with user-defined characteristics of natural history (mean durations of the asymptomatic and symptomatic phases, the duration of the contagious period, the reporting probability, and the characteristics of potential immunity). The generic timeline of the second pathogen is represented on Fig. 1.

#### Second pathogen set-up

If the second pathogen is epidemic, the outbreak set-up is similar to that of influenza, with a mean infectiousness rate, initial immunity proportion, importation period, and number of imported cases all to be defined by the user. If the second pathogen is endemic, a fraction of randomly chosen individuals (20% of the population by default) acquires the pathogen, thus entering the asymptomatic phase at the beginning of the simulation.

### Interactions

Different interaction mechanisms influencing the pathogens’ infection dynamics are embedded in our model. They are based on the main biological mechanisms found in the literature on influenza interactions [2, 7, 8, 13], and summarized as macroscopic mechanisms at the individual level.

**Fig. 2:**
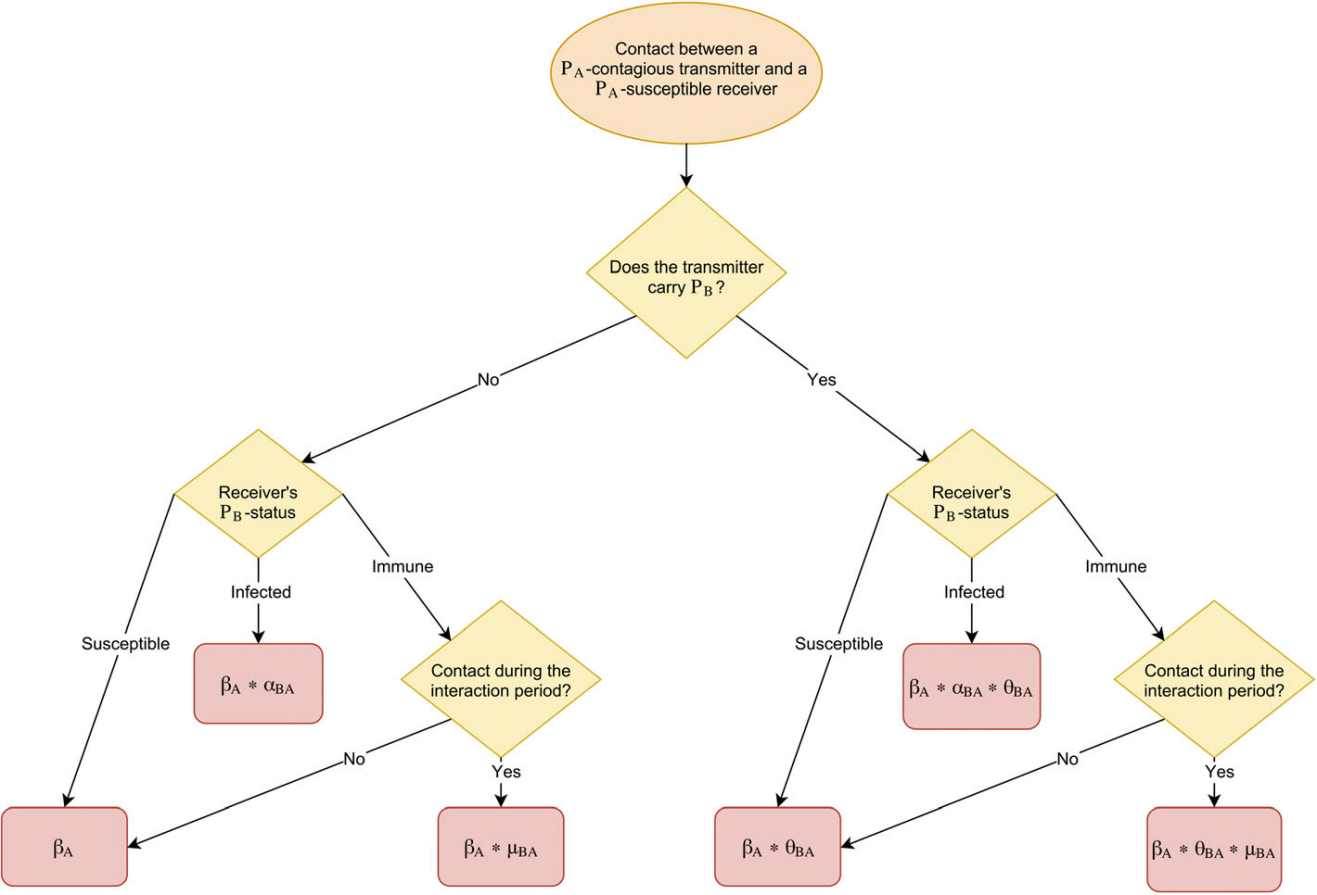
Calculation of a pathogen P_A_’s acquisition probability depending on the two in-contact individuals’ infectious statuses. The transmission probability β_A_ of pathogen P_A_ can be modulated by the different interaction mechanisms, depending on the infectious status of the two individuals in contact. *α*_*BA*_ is the parameter for the acquisition-interaction directed from P_B_ on P_A_, *θ*_*BA*_ is the transmission-interaction parameter and μ_BA_ represents the cross-immunity parameter

The different mechanisms are implemented through multiplicative parameters modulating the pathogens’ transmission (*β*) or infection (p) probabilities (Fig. 2). The “acquisition”-interaction parameter, represented by α, modifies an individual’s probability of actually acquiring a second pathogen when a first one is already present. The “transmission”-interaction parameter, θ, modifies the probability of an individual simultaneously colonized or infected by both pathogens to transmit them to susceptible contacts. The “cross-immunity”-interaction has only been observed in the case of two viruses co-circulating (epidemic pathogens); its parameter, μ, modulates the probability of individuals immune to one pathogen of being infected with the second pathogen. Finally the “pathogenicity”-interaction, with the π parameter, which has only been suggested when an epidemic and an endemic pathogens (eg. bacteria) co-circulate, modifies the probability of developing an infection with an endemic pathogen for individuals carrying both influenza and the endemic pathogen. The modulated probabilities for each interaction phenomenon (except for π which doesn’t occur during transmission) are detailed in Fig. 2.

For all interaction mechanisms, when the interaction parameter is >1, the risk of acquiring, transmitting or developing an infection with a second pathogen is higher for individuals already infected with one pathogen; when the parameter is <1 the risk is lower; and when the parameter = 1, the probability is unchanged and the interaction is not activated. The acquisition, transmission and cross-immunity mechanisms can represent either an influenza impact on the second pathogen’s epidemiology or vice versa. Consequently, for each of these mechanisms, two parameters are defined and can be independently activated or not. For the pathogenicity interaction, we assumed it to be only an effect of influenza on the endemic pathogen, thus only one parameter controls this mechanism. All interaction parameters values are set to 1 by default (Table 1). The duration of the cross-immunity interaction in the pathogen’s cycle can also be specified.

### Use of the model

Through the NetLogo platform we created a user-friendly interface to make our model easy to use and to adapt to test different assumptions or hypotheses [36]. The user is asked to provide values for a list of parameters (default values in Table 1). NetLogo offers different visualization solutions, including plots and variable monitors, while the simulation is running, or recording of chosen variables. By default, our simulator recorded the daily number of new symptomatic cases for influenza and the other pathogen. More outputs can be added easily.

### Stochasticity, reproducibility and technical information

All random numbers and probabilities of transmission, acquisition and infection for both pathogens were randomly chosen. NetLogo uses a random number generator to provide pseudo-random numbers which are determined by the choice of a seed at the beginning of each new simulation.

For the simulations we used the computational and storage services (TARS cluster) provided by the IT Department at Institut Pasteur, Paris. Graphs and statistical analyses were performed with R version 3.3.3.

### Model calibration for the influenza–pneumococcus application

#### Pneumococcus natural history

The pneumococcus parameters default values are given in Table 1, and a timeline of pneumococcal natural history can be found in Additional file 1. Pneumococcus commonly colonizes the human nasopharynx. This colonization state is thought to be mostly asymptomatic and is represented in our model by the asymptomatic period, which lasts an average of 3 weeks [18, 37], after which the bacterium is naturally cleared. During this asymptomatic period, an individual carries the bacteria, is contagious, and at risk of developing a PI. Herein, we considered only pneumococcal pneumonia as a potential PI and retained a 12-day symptomatic period [38], during which the individual is contagious during the first 2 days. In the model, when a subject develops a PI, it is always reported (100% reporting probability assumed), and we considered no immunity to the disease. The carriage rate in the population was set at 20% [17, 39, 40]. The probability of developing a PI was calibrated to obtain an annual average of 220 PI cases per 100,000 [41].

#### Seasonality

PIs have a seasonal trend, with more cases during the winter than summer [42, 43]. We integrated this particularity into the model through a simple threshold function: from October 1st to April 30th, the probability of developing an infection was unchanged, but from May 1st to September 30th this probability was divided by 4, which is consistent with French surveillance data on PIs.

#### Influenza-pneumococcus interactions

Several studies have investigated a potential interaction between influenza virus and pneumococcus [7, 8, 20]. Their results show a unilateral effect of influenza infection on pneumococcus colonization and infection [7, 13, 20], involving several potential biological mechanisms. First, influenza infection is thought to increase the number of binding sites in the nasopharynx leading to increased adherence of the bacterium [8, 13]; at the individual level, it may result in an increased chance of colonization by pneumococcus, modelled here through the acquisition-mechanism. Second, viral infection may increase pneumococcal transmission, potentially as a result of influenza-related symptoms that cause higher shedding of the bacterium [7], this can be directly modelled through the transmission-mechanism. Lastly, influenza infection has been suggested to facilitate the progression from carriage to severe disease [7, 8], an effect modelled here by the pathogenicity-mechanism, which modulates the risk of bacterial disease in colonized (i.e. asymptomatic) individuals (see the figure in Additional file 2).

### Simulations

By varying the values of the different interaction parameters modulating pneumococcal-acquisition (α_ij_), −transmission (θ_ij_), −cross-immunity (μ_ij_), and -pathogenicity (π_ij_), many interaction scenarios can be simulated. We modified independently the parameters corresponding to the influenza-pneumococcus interaction for our simulations: when one interaction mechanism is activated, the corresponding parameter is set to a non-1 value, while the other interaction parameters are fixed to 1. For each of the three parameters considered here (α_12_, θ_12_, and π_12_), the following values were independently tested: 2, 5, 10, 15, 25, 50, 75, 100, corresponding to a total of 24 scenarios. A baseline scenario with no interaction was also simulated. For each scenario, 1000 independent epidemiological years were simulated from the model.

### Evaluation of PI burden

The ABM structure operates at the individual level, offering the possibility to monitor each person’s history for both pathogens. The exact PI-case count caused by the influenza interaction can then be derived, and the influenza-related burden of PI computed. As the acquisition- and the transmission-interaction mechanisms operate during pneumococcal carriage, some PIs are directly caused by the interaction between the virus and the bacterium, while others are secondary cases that are an indirect consequence of the interaction. For example, someone who acquired pneumococcus as a result of the interaction and then developed a PI is a direct PI case, but if this person transmits pneumococcus to someone else and this second individual then develops a PI, it is an indirect PI case. Because the pathogenicity mechanism operates during the transformation from carrier to infected individual, it only leads to direct cases per our definition.

The influenza-induced burden of PIs for one scenario was calculated as the difference between the mean total number of PIs for the considered scenario (over the 1000 iterations) and the mean total number of PIs for the baseline scenario in which no influenza–pneumococcus interaction mechanism was activated. The burden was assessed for three different periods of the simulated epidemiological year: during influenza season only, post-influenza season only, and during the entire year (therefore including the two previously cited periods). The influenza season is defined as weeks with >150 new influenza cases reported, the post-influenza season is the period starting immediately after the end of the influenza season, as we defined it, and lasting until the end of the simulated year (September 30th).

## Results

In the following, all average numbers of cases are reported per 100,000 inhabitants.

### Model outputs

#### Simulation of influenza

**Fig. 3:**
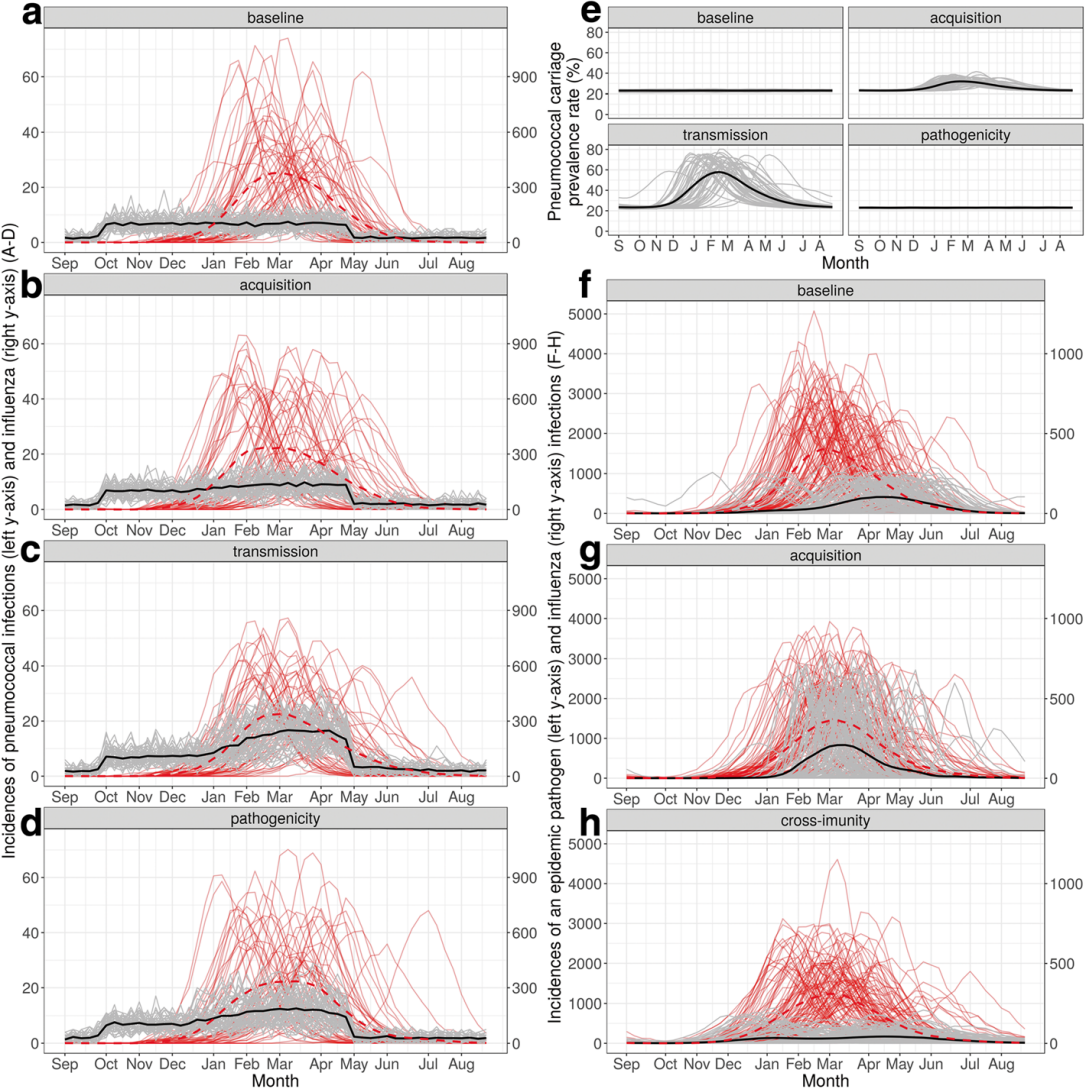
Weekly incidence of simulated cases per 100,000 for influenza and the two possible co-circulating pathogens. 50 iterations of the model (grey and red lines) are presented, along with the average incidence over those iterations (black line for PI or second epidemic pathogen, dashed red line for influenza). (**a**–**d**) For influenza (red), the following parameter values were used for the simulations: transmission probability 3.3% per contact-day; 23% of the population initially immunized; 20% case-reporting probability; no interaction mechanism activated between influenza and the second pathogen. For PI cases (black and grey), the following parameter values were used: carriage rate 20%; pathogenicity rate 4.2e-5 per day; no immunity; 100% case-reporting probability; no interaction mechanism activated (**a**), acquisition-interaction strength 50 (**b**), transmission-interaction strength 50 (**c**), and pathogenicity-interaction strength 50 (**d**). (**e) ** Pneumococcal carriage prevalence rate for the baseline scenario, the acquisition-interaction strength 50, the transmission-interaction strength 50, and the pathogenicity-interaction strength 50. (**f**–**h**) For influenza (red) and a second epidemic pathogen (black and grey) cases, the following parameter values were used for the latter: transmission probability 2.8% per contact-day; 25% of the population initially immunized; 20% case-reporting probability; no interaction mechanism activated (**f**), acquisition-interaction strength 25 (**g**), and cross-immunity–interaction strength 0.8 (**h**)

Influenza dynamics are well-reproduced by the model, as displayed in Fig. 3 and the figure in Additional file 3. The mean duration of each outbreak and the average maximum numbers of influenza cases per year are all consistent with reported incidence data in France, extrapolated from the Sentinel Network [35]. Variations among the different years in terms of timing and size of the epidemics are visible, due to the stochasticity of the model. On average, over 1000 iterations, we found 5260 ([3760–6760] 95% confidence interval) annual influenza cases.

#### Simulation of an endemic pathogen (pneumococcus)

Weekly incidence series for PIs are presented in Fig. 3a–d. We reproduced several hundred PIs per year (mean 242 cases). The majority of PIs occurred in the winter (mean 203 from October to April), which is in accordance with the pneumococcal pneumonia incidence in France [44]. As observed in Fig. 3a–d and Additional file 3, the PI-incidence series differed markedly depending on which interaction mechanism was activated. In particular, pneumococcus prevalence (Fig. 3e) strongly shadowed the influenza outbreak for the acquisition and transmission mechanisms.

#### Simulation of another epidemic pathogen

Figure 3f–h and Additional file 3 illustrate the two successive epidemic series occurring during successive simulated years for different interaction mechanisms from influenza on a second epidemic pathogen. Each mechanism specifically altered the resulting shape and timing of the second pathogen’s outbreak. Strong over-induced acquisition (Fig. 3g) generated dramatically higher numbers of infections for the second epidemic pathogen (interaction-parameter strength 50). For strong cross-immunity interaction (interaction-parameter strength 0.8), the second epidemic was delayed several weeks (Fig. 3h), and the total number of cases was much lower than for the baseline scenario (Fig. 3f).

### Influenza-related PI burden

In our simulations, the average influenza season lasted 11.6 weeks, and the post-influenza period lasted 26.6 weeks.

#### Baseline scenario

As expected, an average annual number of 242 PIs (mean 4.6 per week) was estimated when no between-pathogen interaction was considered [see the table in Additional file 4]. In accordance with pneumococcal seasonality, higher average numbers of cases occurred during the influenza season: 6.2 cases per week (72 cases in 11.6 weeks). In contrast, a mean of 2.8 cases per week, corresponding to 75 cases in 26.6 weeks, were obtained after the influenza season.

#### Influenza-induced over-acquisition

**Fig. 4:**
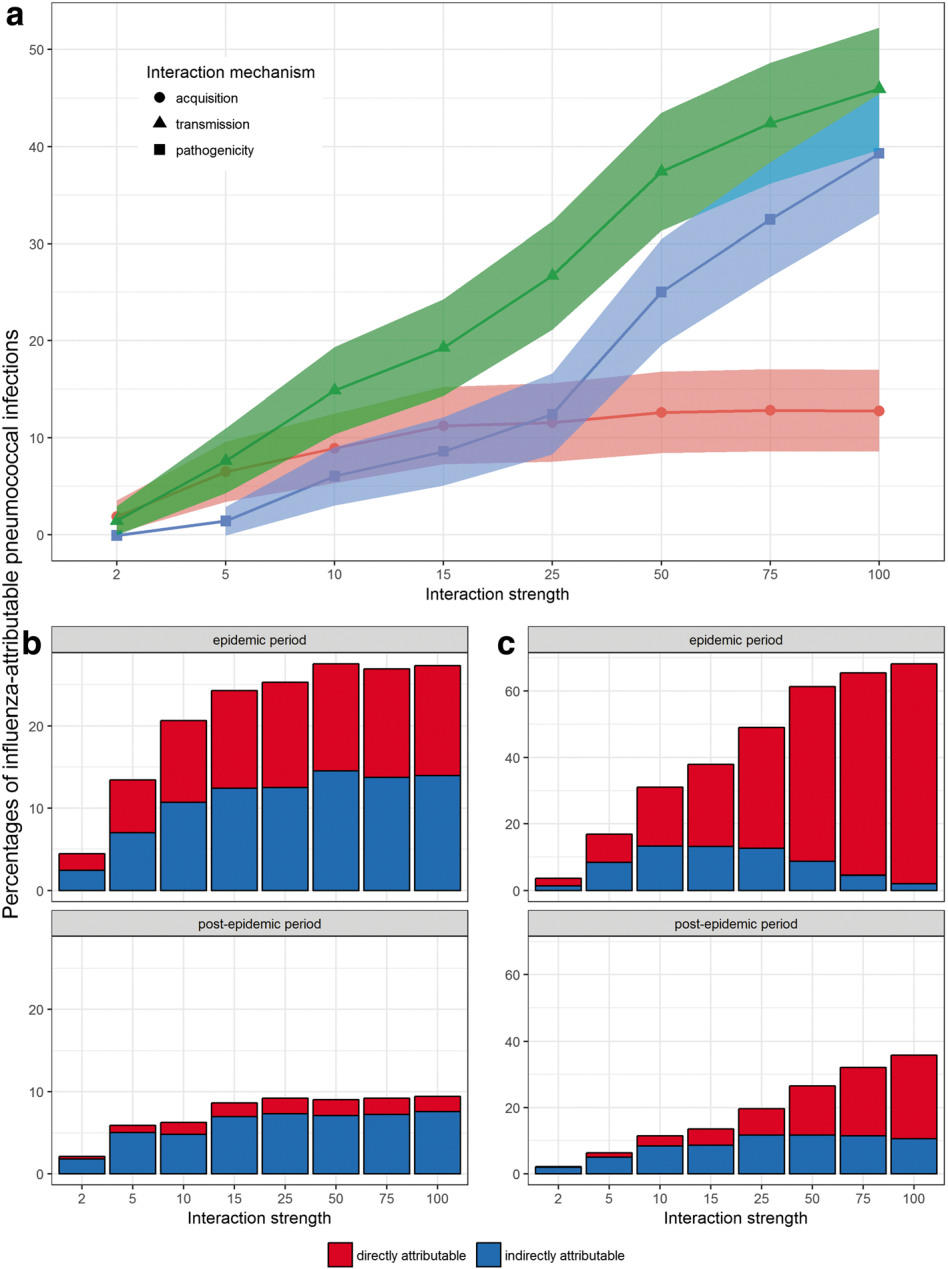
Computed influenza-induced PI burden**.** Average percentages and 95% confidence intervals of influenza-attributable PIs over the 1000 iterations of the model (y-axis), according to the acquisition-, transmission- or pathogenicity-interaction mechanism, and for the range of interaction-strength values tested (x-axis). Global burden of PIs over the entire simulated year (**a**), direct and indirect number of PIs for the (**b**) acquisition-interaction mechanism or (**c**) transmission-interaction mechanism

The burden of influenza-attributable PIs increased slightly with the acquisition parameter: above the threshold value of 15 it remained stable, representing on average 34 PIs out of 276 annual cases (around 12% of the total number of PIs) for the whole year, even for very high interaction-parameter values (Fig. 4a). During the influenza period, the influenza-induced PI burden also showed a threshold interaction-parameter value of 15, with an average burden of 26% of all PIs during this period (Fig. 4b – upper panel and Additional file 4). During the weeks following the influenza season, the interaction effect was prolonged with persistently about 9% of all that period’s PIs which can be attributed to influenza for any parameter value (Fig. 4b – lower panel and Additional file 4). However, most of those PIs resulted from indirect/secondary cases and only 2% came from cases directly attributable to the interaction.

#### Influenza-induced over-transmission

The burden of influenza-attributable PI cases increased with the transmission-related parameter, regardless of the time period (whole year, influenza season or post-influenza season), reaching an influenza-induced burden of 68% for the highest parameter value during the influenza season (Fig. 4c – upper panel). The same held true for the relative numbers of directly attributable cases. Interestingly, when the interaction parameter increased, more direct than indirect cases occurred.

#### Influenza-induced increased pathogenicity

The burden of influenza-attributable PI cases for the pathogenicity interaction was lower than the one for the acquisition interaction for parameter values up to 15, and lower than the PI burden for the transmission interaction for parameter values up to 100 (Fig. 4a). However the burden associated with the pathogenicity mechanism regularly increased with the interaction strength, the more striking increase being during the influenza season when it reached 65% of all PI cases for the strongest interaction link [see Additional file 4].

#### Timing of the interaction mechanisms

The effects of the different interaction mechanisms on the PI burden do not immediately follow the introduction of influenza, this lag was investigated by calculating the mean weekly burden for each interaction scenario. The results are exposed in the figure in Additional file 5. On one hand, a significant burden impact of the pathogenicity-interaction is generally observed 2 to 5 weeks after the first cases of influenza are introduced in the population. On the other hand, consequences of the acquisition- and transmission-interaction mechanisms are observed after a longer delay, and have longer-lasting effects on the influenza-induced burden than the pathogenicity mechanism, due to their impact on colonization.

## Discussion

We developed a novel simulator of influenza virus in interaction with a second pathogen. The SimFI framework successfully reproduces the co-circulation of influenza and other pathogens, either epidemic or endemic. We obtained realistic simulated datasets in terms of weekly incidence, epidemic size and timeframe, and carriage rate. The effects of the different influenza–pneumococcus-interaction mechanisms implemented were assessed. For the endemic pathogen, the effects of the acquisition and transmission mechanisms on the dynamics were similar and could not be discriminated visually on the incidence series; the pathogenicity mechanism also had similar dynamics but the incidence series more closely paralleled the influenza epidemics than for the former two.

We used the SimFI framework to evaluate the influenza-attributable PI burden in a series of simulations, characterized by different interaction hypotheses. We found that for the acquisition-interaction mechanism, the burden rapidly reached a maximum around 26% of PIs during the influenza period, when the interaction strength increased (threshold reached for interaction strengths >10). The transmission- and pathogenicity-interaction mechanisms behaved differently. The PI burden increased with the transmission-interaction–parameter value for the three considered periods (entire year, influenza season, and post-influenza season), with respective values in the ranges 1–52%, 3–72%, and 2–46% of total PIs. Same was observed for the pathogenicity mechanism, with respective average PI burdens in the ranges of 1–56%, 1–79%, and 0–24%.

Herein, we tested a wide range of strengths for the influenza-virus–pneumococcus interaction (applied to pneumococcal pneumonia) with interaction-parameter values ranging from 2 to 100, in accordance with the estimations of previous modeling studies for different types of pneumococcal infections [28, 45]. Based on French pneumococcal meningitis cases over 2001–2004, Opatowski et al. [28] found transmissibility increased by a factor of 8.7 for co-infected individuals and pathogenicity by 92. Based on two distinct US pneumonia datasets, Shrestha et al. [45] found that influenza infection increased susceptibility to pneumococcal pneumonia by factors 85 and 115. We note that, due to differences in model structures, the susceptibility-interaction parameter mentioned in their study corresponds to a combination of the acquisition- and pathogenicity-interaction parameters from our model. The values used for our simulations were intentionally chosen to include the values estimated in those earlier studies. It is important to note that some parameter values, particularly the high acquisition- and transmission-interaction values (Fig. 3e), resulted in strong seasonality of pneumococcal carriage. That phenomenon is not reported in literature, which suggests that these scenarios might not be realistic.

An advantage of the SimFI framework is the possibility to exactly count the number of infections with each pathogen, as well as the exact number of cases attributable to the between-pathogen interaction. The authors of several studies attempted to estimate the PI burden. Classically, burden estimations have been based on regression models, including influenza as a covariate, and comparison to a baseline deduced by removing influenza. For example, Walter et al. [46] estimated the influenza-induced burden of invasive pneumococcal pneumonia in the United States, and found that 11%–14% of those pneumonia cases could be attributed to influenza during the influenza season, and 5%–6% over the year. Those estimations are in accordance with some of the parameter values used in our model: an over-acquisition strength around 5, an over-transmission strength between 2 and 5, or an increased pathogenicity strength between 5 and 10 are all compatible with Walter’s results. In the study by Nicoli et al. [47] on United Kingdom surveillance data, 7.5% of invasive pneumococcal diseases were estimated to be attributable to influenza over the year, consistent with several of our scenarios: acquisition increased by 5–10, transmission increased by 5, or pathogenicity increased by 10–15. However, the estimation from real incidence data of the number of excess infection cases caused by between-pathogens interactions is difficult, because there is no direct information on infection history of co-circulating pathogens. Furthermore, no consensus has yet been reached on the method to use to estimate the associated burden, complicating the comparison between the different studies’ results on this topic.

Herein, we aimed to formalize the simplest model incorporating the main features of influenza interactions with another pathogen. Our model is therefore a simplification of reality. A sensitivity analysis was carried out on several key parameters: pneumococcus carriage rate, mixing patterns in the simulated world, and size of the influenza epidemic. The movement patterns of the individuals did not affect the influenza-induced PI burden, however both the carriage rate of pneumococcus in the population and the size of the influenza epidemic had an impact on the PI burden (results available in Additional file 6). More complex features could be included, for example by incorporating an age structure that would allow modeling of acquired immunity for one or both pathogens. That inclusion would be particularly relevant for the influenza–pneumococcus interaction model, given that pneumococcal carriage varies markedly with respect to the age of individuals [17], and that influenza disease and PI severity have also been suggested to be age-related [16, 48]. Furthermore, the simulator could be extended to test other interaction hypotheses in addition to the ones we implemented: for example, if we were to allow individuals to die from illness, we could integrate an interaction for disease severity. The SimFI model parameters would also be easy to adapt to test interactions for an agent other than influenza, and then implement completely different interaction mechanisms.

The model we propose could also be used and extended to simulate and predict the effects of public health measures targeting one or two pathogens. That extension would allow the assessment of such measures by taking a broader view, including co-circulating pathogens. The impact of vaccinating against influenza on PIs could for example be assessed, or the effect of more complex scenarios when two or more control measures are implemented simultaneously (e.g. simultaneous vaccinations against influenza and pneumococcus).

## Conclusions

In conclusion, a novel individual-based simulator of influenza in interaction with another pathogen, including several ready-to-use interaction mechanisms, was described. As an application, we used this SimFI framework to assess what the influenza-associated PI burden could be for different hypothetical interactions. This open-access model will provide new opportunities to test hypotheses concerning pathogen (especially influenza) interactions, generate in silico data for different scenarios, and assess the statistical methods generally used to study interactions.

## Additional files

Additional file 1: Natural histories of influenza and pneumococcus. Clinical status (upper rectangles) and infectious status (green for non-contagious, red for contagious) are shown for influenza (top timeline) and pneumococcus (bottom timeline). (JPEG 72 kb)

Additional file 2: Calculation of pneumococcus’ acquisition probability depending on the two in-contact individuals’ infectious statuses. The transmission probability β_Sp_ of pneumococcus (*Streptococcus pneumoniae*) can be modulated by the different interaction mechanisms, depending on the infectious status of the two individuals in contact. *α*_*12*_ is the parameter for the acquisition-interaction directed from influenza on pneumococcus, and *θ*_*12*_ is the transmission-interaction parameter. (PNG 67 kb)

Additional file 3: Weekly incidence of simulated cases per 100,000 for the three possible pathogens. (A–D) For influenza (red), the following parameter values were used for the simulations: transmission probability 3.3% per contact-day; 23% of the population initially immunized; 20% case-reporting probability; no interaction mechanism activated between influenza and the second pathogen. For PI cases (black), the following parameter values were used: carriage rate 22% per contact-day; pathogenicity probability 0.0042% per day; no immunity; 100% case-reporting probability; no interaction mechanism activated (A), acquisition-interaction strength 50 (B), transmission-interaction strength 50 (C), and pathogenicity-interaction strength 50 (D). (E) Pneumococcal carriage prevalence for the baseline scenario (orange), the acquisition-interaction strength 50 (green), the transmission-interaction strength 50 (blue), and the pathogenicity-interaction strength 50 (purple); (F–H) For influenza (red) and a second epidemic pathogen (black) cases, the following parameter values were used for the latter: transmission probability 2.8% per contact-day; 25% of the population initially immunized; 20% case-reporting probability; no interaction mechanism activated (F), acquisition-interaction strength 25 (G), and cross-immunity–interaction strength 0.8 (H). The represented data were chosen for five among the 1000 simulated years for each scenario for their explicit representation of each interaction-mechanism effect on infection dynamics. (PNG 424 kb)

Additional file 4: Burden of influenza-related pneumococcal infections for all simulated scenarios. Eight different values of interaction parameters were tested for each interaction mechanism (acquisition, transmission, and pathogenicity). Values in the table represent the influenza-induced excess numbers of pneumococcal infections and the associated percentages, presented with their 95% confidence intervals, per 100,000. Values are aggregated over the three different periods considered: whole epidemiological year, influenza season, and post-influenza season. (XLSX 16 kb)

Additional file 5: Weekly influenza-induced PI burden after the introduction of influenza in the population. The average weekly burdens for different scenarios are represented if they are statistically significant (non-0 values). The x-axis represents the number of weeks after the introduction of influenza in the population. (PNG 213 kb)

Additional file 6: Sensitivity analysis of pneumococcus carriage rate, movement patterns in the population, and influenza size. (PDF 181 kb)

## Abbreviations

ABM: Stands for agent-based model
PI: Stands for pneumococcal infections
SimFI: Stands for simulator of influenza in interaction
