# Supplementary figures and images for: An agent-based model simulation of influenza interactions at the host level: insight into the influenza-related burden of pneumococcal infections
Source: BMC Infect Dis. 2017 Jun 2;17:382. doi: 10.1186/s12879-017-2464-z (PMC5455134; doi:10.1186/s12879-017-2464-z)

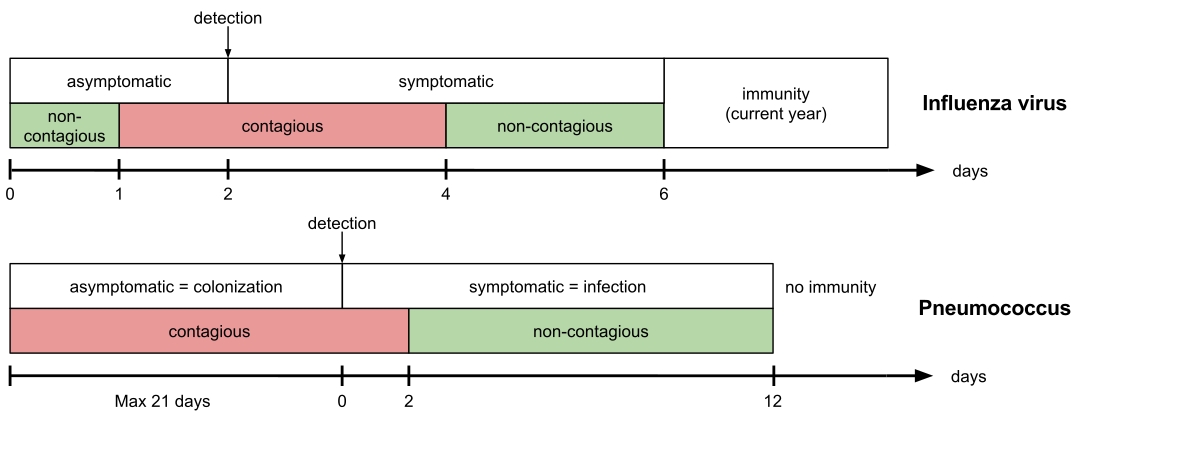

Supplement: Supplementary file 1 — Natural histories of influenza and pneumococcus. Clinical status (upper rectangles) and infectious status (green for non-contagious, red for contagious) are shown for influenza (top timeline) and pneumococcus (bottom timeline). (JPEG 72 kb) [file 12879_2017_2464_MOESM1_ESM.jpg]

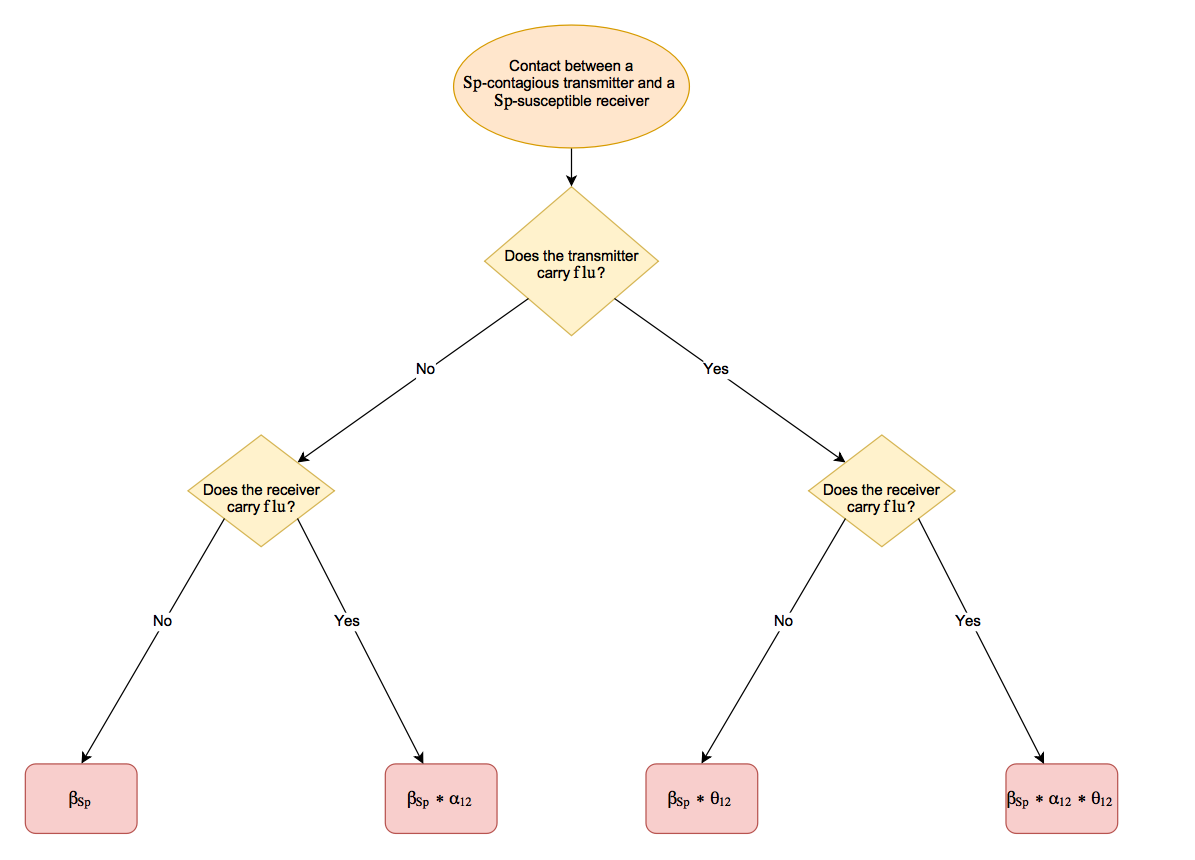

Supplement: Supplementary file 2 — Calculation of pneumococcus’ acquisition probability depending on the two in-contact individuals’ infectious statuses. The transmission probability βSp of pneumococcus (Streptococcus pneumoniae) can be modulated by the different interaction mechanisms, depending on the infectious status of the two individuals in contact. α 12 is the parameter for the acquisition-interaction directed from influenza on pneumococcus, and θ 12 is the transmission-interaction parameter. (PNG 67 kb) [file 12879_2017_2464_MOESM2_ESM.png]

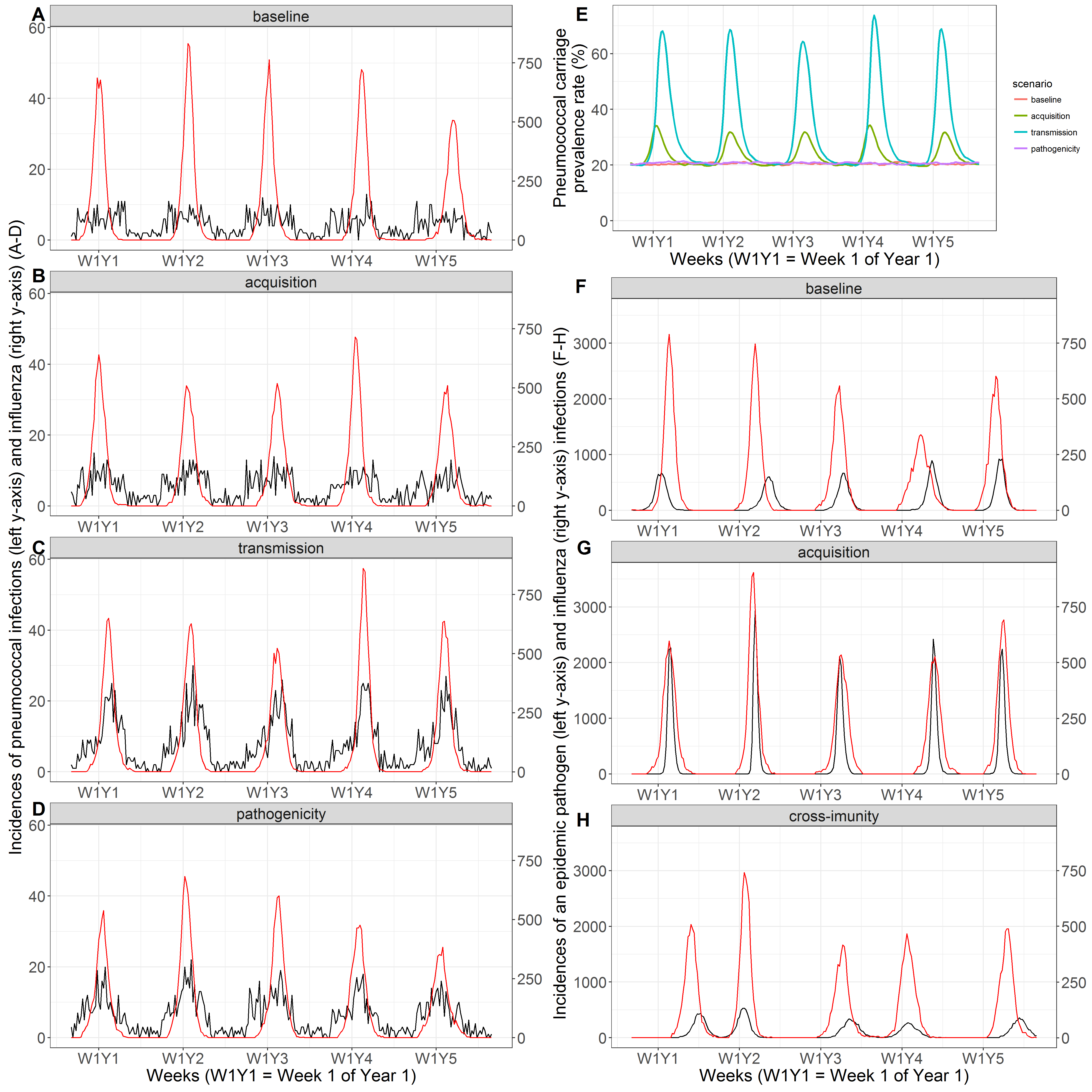

Supplement: Supplementary file 3 — Weekly incidence of simulated cases per 100,000 for the three possible pathogens. (A–D) For influenza (red), the following parameter values were used for the simulations: transmission probability 3.3% per contact-day; 23% of the population initially immunized; 20% case-reporting probability; no interaction mechanism activated between influenza and the second pathogen. For PI cases (black), the following parameter values were used: carriage rate 22% per contact-day; pathogenicity probability 0.0042% per day; no immunity; 100% case-reporting probability; no interaction mechanism activated (A), acquisition-interaction strength 50 (B), transmission-interaction strength 50 (C), and pathogenicity-interaction strength 50 (D). (E) Pneumococcal carriage prevalence for the baseline scenario (orange), the acquisition-interaction strength 50 (green), the transmission-interaction strength 50 (blue), and the pathogenicity-interaction strength 50 (purple); (F–H) For influenza (red) and a second epidemic pathogen (black) cases, the following parameter values were used for the latter: transmission probability 2.8% per contact-day; 25% of the population initially immunized; 20% case-reporting probability; no interaction mechanism activated (F), acquisition-interaction strength 25 (G), and cross-immunity–interaction strength 0.8 (H). The represented data were chosen for five among the 1000 simulated years for each scenario for their explicit representation of each interaction-mechanism effect on infection dynamics. (PNG 424 kb) [file 12879_2017_2464_MOESM3_ESM.png]

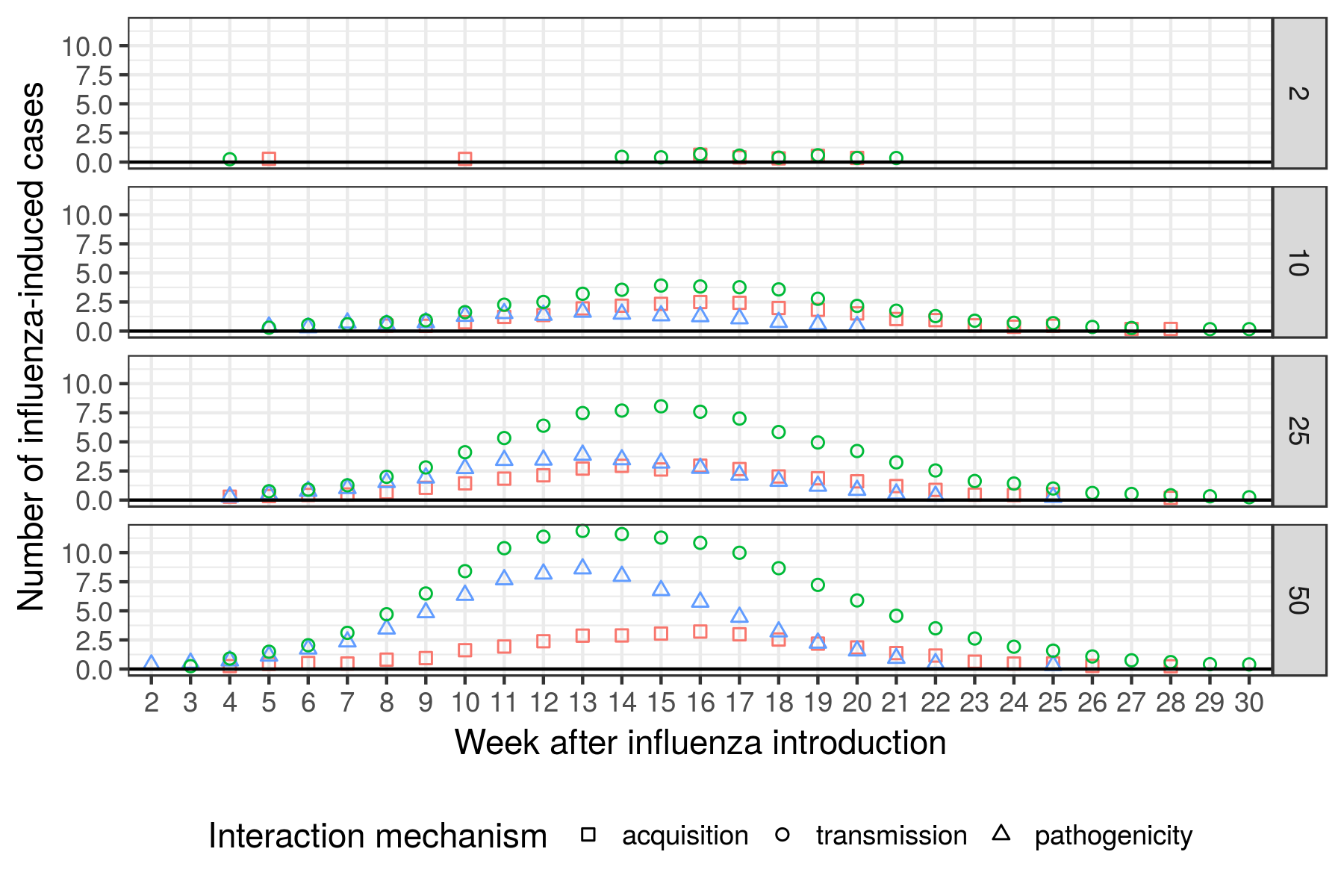

Supplement: Supplementary file 5 — Weekly influenza-induced PI burden after the introduction of influenza in the population. The average weekly burdens for different scenarios are represented if they are statistically significant (non-0 values). The x-axis represents the number of weeks after the introduction of influenza in the population. (PNG 213 kb) [file 12879_2017_2464_MOESM5_ESM.png]
