# Supplementary material for: An agent-based model simulation of influenza interactions at the host level: insight into the influenza-related burden of pneumococcal infections
Source: BMC Infect Dis. 2017 Jun 2;17:382. doi: 10.1186/s12879-017-2464-z (PMC5455134; doi:10.1186/s12879-017-2464-z)

Figure 6.1. Number of influenza-attributable PI cases for different carriage rates of pneumococcus in the population. The average burden over the simulated years was assessed for three different values of pneumococcus' carriage rate (10%, 20%, and 30%), for a selection of scenarios. We note that because the pathogenicity mechanism involves a linear process, even if the number of cases increases with the carriage rate, the proportion of influenza-attributable PI remains constant.

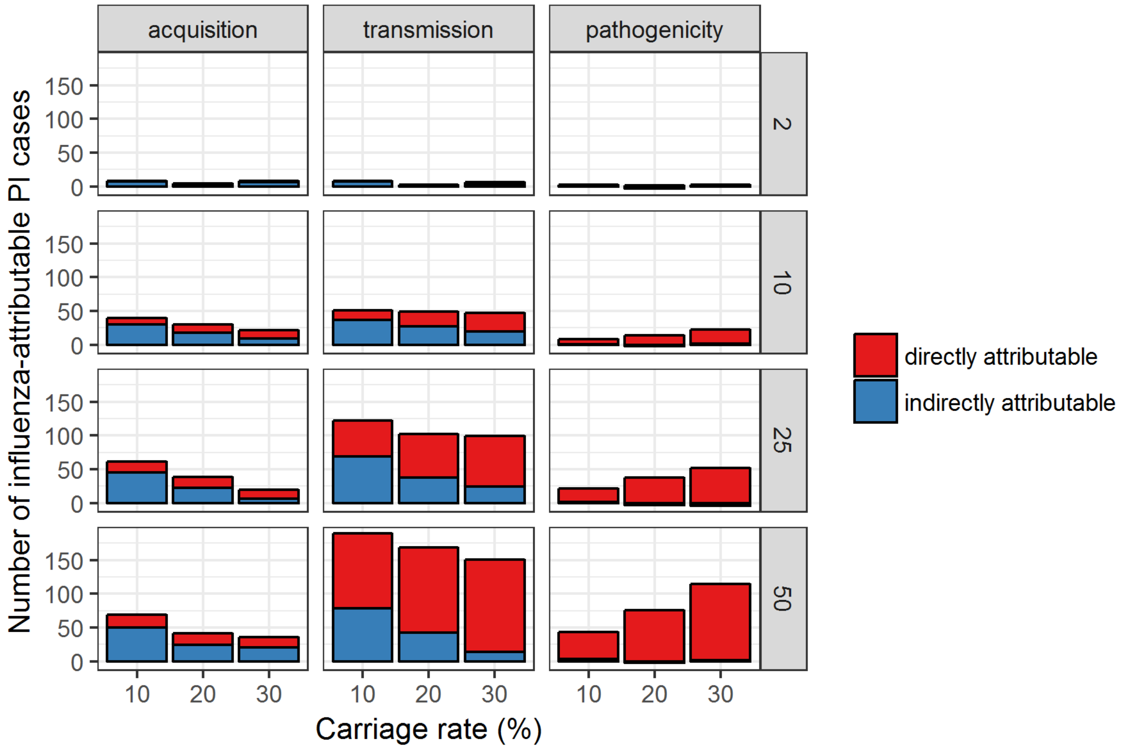

Figure 6.2. Number of influenza-attributable PI cases for different movement patterns of the individuals in the simulated world. The average burden over the simulated years was assessed for three different values of "jump sizes" representing the movements of the individuals around the simulated world (2, 10, 50), for a selection of scenarios.

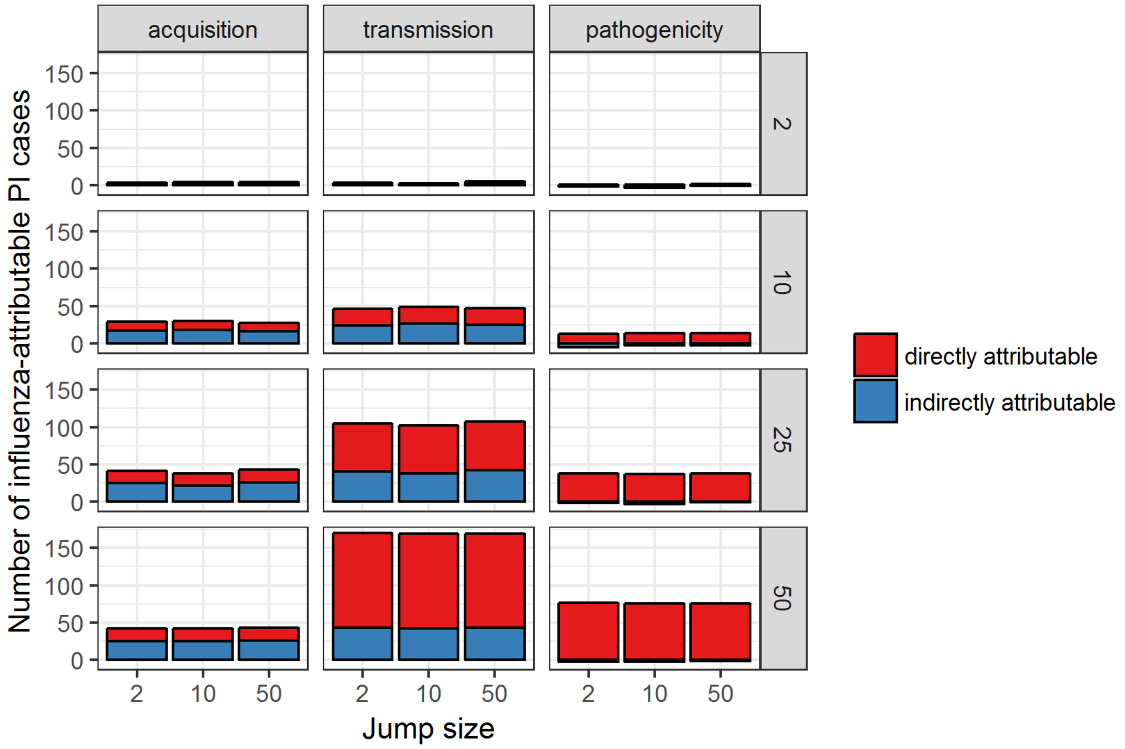

Figure 6.3. Influenza-induced burden of PI cases depending on the type of influenza epidemic. Seasonal influenza epidemics corresponding to R0=1.3, and pandemic influenza outbreaks corresponding to R0=2 were simulated. The average burden associated with each type of influenza epidemic is represented for the three interaction mechanisms.

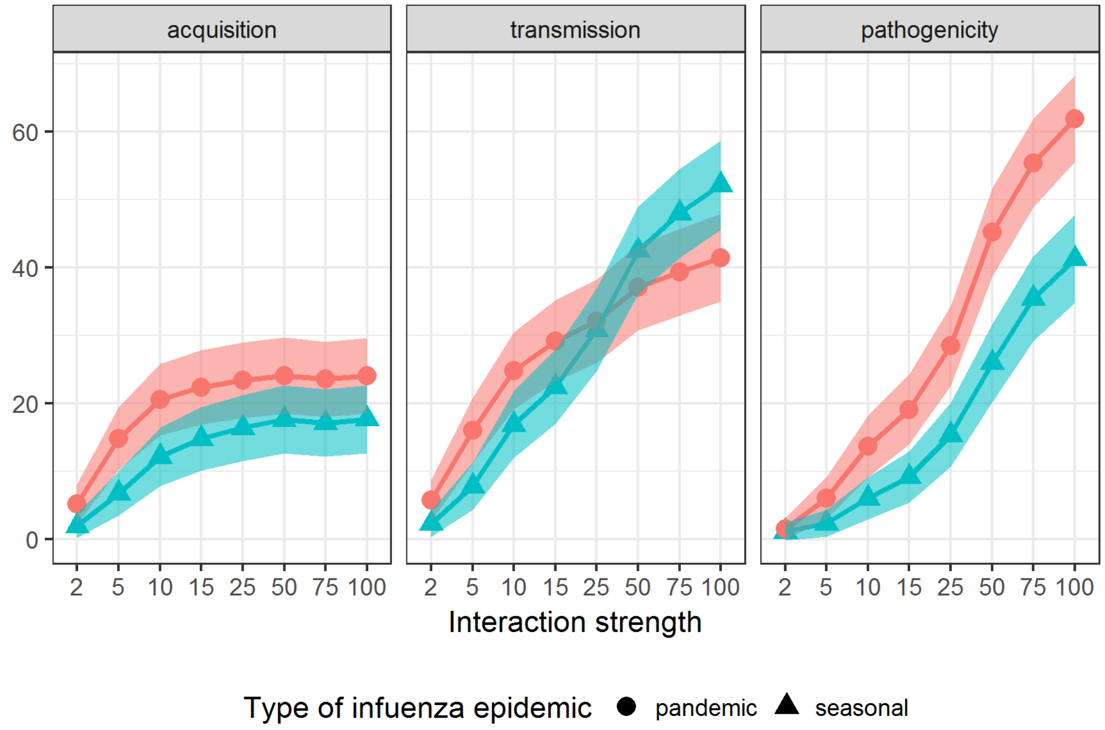

Supplement: Supplementary file 6 — Sensitivity analysis of pneumococcus carriage rate, movement patterns in the population, and influenza size. (PDF 181 kb) [file 12879_2017_2464_MOESM6_ESM.pdf]
